# Enzalutamide Sensitizes Castration‐Resistant Prostate Cancer to Copper‐Mediated Cell Death

**DOI:** 10.1002/advs.202401396

**Published:** 2024-06-10

**Authors:** Xiang Gao, Haolin Zhao, Jiao Liu, Min Wang, Zhihong Dai, Wenjun Hao, Yanlong Wang, Xiang Wang, Min Zhang, Pixu Liu, Hailing Cheng, Zhiyu Liu

**Affiliations:** ^1^ Department of Urology Second Hospital of Dalian Medical University Dalian 116023 China; ^2^ Dalian Key Laboratory of Molecular Targeted Cancer Therapy Cancer Institute The Second Hospital of Dalian Medical University Dalian 116023 China; ^3^ Zhejiang Key Laboratory of Intelligent Cancer Biomarker Discovery and Translation The First Affiliated Hospital of Wenzhou Medical University Wenzhou 325000 China; ^4^ Liaoning Engineering Research Center of Integrated Precision Diagnosis and Treatment Technology for Urological Cancer Dalian 116023 China

**Keywords:** copper ionophore, CRPC, cuproptosis, enzalutamide

## Abstract

Despite the initial efficacy of enzalutamide in castration‐resistant prostate cancer (CRPC), inevitable resistance remains a significant challenge. Here, the synergistic induction of copper‐dependent cell death (cuproptosis) in CRPC cells is reported by enzalutamide and copper ionophores (elesclomol/disulfiram). Mechanistically, enzalutamide treatment increases mitochondrial dependence in CRPC cells, rendering them susceptible to cuproptosis, as evidenced by specific reversal with the copper chelator tetrathiomolybdate. This susceptibility is characterized by hallmarks of cuproptosis, including lipoylated protein aggregation and iron‐sulfur cluster protein instability. Interestingly, the mitochondrial matrix reductase, FDX1, specifically correlates with elesclomol sensitivity, suggesting a potential mechanistic divergence between the two copper ionophores. Notably, this synergistic effect extends beyond in vitro models, demonstrating efficacy in 22Rv1 xenografts, mouse *Pten p53* knockout organoids. Importantly, enzalutamide significantly enhances copper ionophore‐mediated cytotoxicity in enzalutamide‐resistant cells. Collectively, these findings indicate that enzalutamide and copper ionophores synergistically induce cuproptosis, offering a promising therapeutic avenue for CRPC, potentially including enzalutamide‐resistant cases.

## Graphical abstract

This study uncovers a strong synergy between enzalutamide and copper ionophores, resulting in cuproptosis (copper‐dependent cell death) in castration‐resistant prostate cancer (CRPC) cells. The heightened dependence on mitochondria following enzalutamide treatment increases susceptibility to cuproptosis. Effectiveness is demonstrated across diverse models, including CRPC cell lines, xenografts, enzalutamide‐resistant models, and mouse prostate organoids, highlighting the translational potential of these findings.

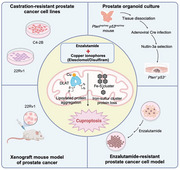

## 1 Introduction

Castration‐resistant prostate cancer (CRPC) represents an aggressive and advanced stage of the disease characterized by persistent tumor growth despite therapeutic testosterone destruction through androgen deprivation therapy (ADT).^[^ ^1^, ^2^ ^]^ The resistance to hormonal control and the associated poor prognosis highlights the urgent need for innovative treatment strategies. Enzalutamide (ENZ), a second‐generation androgen receptor inhibitor, initially showed promise for CRPC patients. However, resistance inevitably develops, driven by mechanisms such as androgen receptor mutations, gene amplification, and activation of alternative signaling pathways.^[^ ^3^, ^4^ ^]^ Intensive research efforts are focused on elucidating the mechanisms of resistance and developing innovative anticancer strategies to combat this aggressive and drug‐resistant disease.

Emerging evidence reveals a complex relationship between copper dysregulation and the progression of prostate cancer.^[^ ^5^, ^6^, ^7^, ^8^ ^]^ Prostate cancer patients have been reported to exhibit increased levels of serum copper, enhanced cellular uptake, and androgen‐mediated intracellular copper accumulation, strongly suggesting copper dysregulation as a potential hallmark of the disease. The discovery of cuproptosis as a distinct, copper‐dependent cell death pathway opens the door for the development of innovative cancer therapies.^[^ ^9^, ^10^, ^11^, ^12^ ^]^ Copper ionophores, such as elesclomol and disulfiram, exploit this vulnerability by selectively targeting cancer cells with elevated copper uptake and inducing cuproptosis.^[^ ^9^ ^]^ Despite promising preclinical activity,^[^ ^13^, ^14^ ^]^ prior clinical trials with elesclomol and disulfiram have not yet translated into patient benefits in unselected populations, including those with prostate cancer (clinicaltrials.gov, NCT00808418, NCT01118741, NCT02963051). This underscores the need for further investigation and optimization of copper‐based therapies, particularly in therapy‐resistant CRPC patients.

Cancer cells that are highly dependent on mitochondrial respiration exhibit increased susceptibility to copper ionophores.^[^ ^9^, ^15^ ^]^ Exploiting this metabolic vulnerability by inducing cuproptosis represents a promising avenue for therapeutic intervention in prostate cancer. Notably, recent studies have shown that AR blockade by enzalutamide reprograms cellular metabolism in prostate cancer cells, resulting in an increased reliance on mitochondrial oxidative phosphorylation (OXPHOS).^[^ ^16^, ^17^ ^]^ In this study, our independent findings add to this understanding by demonstrating that enzalutamide treatment induces prostate cancer cells to become dependent on oxidative phosphorylation. We investigated the potential of combining ENZ with copper ionophores as a novel therapeutic strategy for CRPC. We hypothesize that ENZ‐induced dependence on OXPHOS renders CRPC cells more susceptible to copper‐mediated cell death induced by elesclomol and disulfiram. By elucidating the underlying mechanisms and validating the efficacy in multiple preclinical models, this study aims to pave the way for a promising new approach to combat CRPC, potentially including ENZ‐resistant cases.

## 2 Results

### 2.1 Copper Ionophores Mediated Cytotoxicity in Prostate Cancer Cells

**Figure 1:**
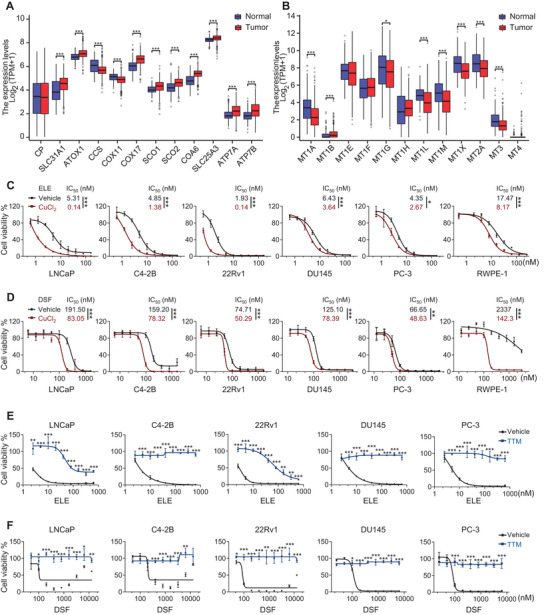
Copper ionophores induced cytotoxicity in prostate cancer cells. Differences in the expression levels of A) 12 genes involved in regulating copper homeostasis and B) 12 metallothionein genes between tumor and normal samples in TCGA_PRAD datasets. TCGA, The Cancer Genome Atlas. PRAD, Prostate adenocarcinoma. Tumor, red; Normal, blue. ^*^ *P* < 0.05, ^***^ *P* < 0.001 (Wilcoxon rank sum test). The viability of cell lines as indicated was measured by crystal violet assay after treatment with C) elesclomol (ELE) or D) disulfiram (DSF), with or without 1 µM CuCl_2_ for 5–7 days. Data are shown as Mean ± S.D. for three independent experiments. ^*^ *P* < 0.05, ^**^ *P* < 0.01, ^***^ *P* < 0.001 (two‐way ANOVA with Bonferroni's Tukey's multiple comparisons test). The viability of cells as indicated was measured by crystal violet assay after treatment with E) ELE or F) DSF, with or without 2 µM TTM for 5–7 days. Data are shown as Mean ± S.D. for three independent experiments. ^*^ *P* < 0.05, ^**^ *P* < 0.01, ^***^ *P* < 0.001 (one‐way ANOVA, with Tukey's multiples comparison test).

Dysregulation of copper homeostasis (uptake, storage, and utilization) has emerged as a prominent feature of prostate cancer.^[^ ^14^, ^18^, ^19^ ^]^ Analysis of TCGA prostate cancer data revealed dysregulation of key molecular pathways regulating copper homeostasis (**Figure** **1**A), evidenced by altered expression patterns in genes governing copper acquisition, trafficking, storage, and export.^[^ ^18^ ^]^ Notably, downregulation of a substantial fraction of metallothionein proteins, crucial for copper storage,^[^ ^20^ ^]^ was observed in tumor tissues (Figure 1B), indicating impaired copper buffering capacity in prostate cancer cells. These results reveal a potential vulnerability to copper‐induced stress due to dysregulated copper metabolism. Further supporting this vulnerability, prostate cancer cells exhibited significantly increased sensitivity to the copper ionophores elesclomol (ELE) and disulfiram (DSF) compared to normal cells (Figure 1C,D). This selective cytotoxicity is most likely mediated by intracellular copper accumulation,^[^ ^9^, ^10^ ^]^ as exogenous copper supplementation with CuCl_2_ further potentiated the cytotoxicity of ELE and DSF (Figure 1C,D). Importantly, the copper chelator tetrathiomolybdate (TTM) readily reversed the inhibitory effects of ELE and DSF (Figure 1E,F), validating copper accumulation as the underlying mechanism of their cytotoxicity. Collectively, these findings underscore the therapeutic potential of targeting copper homeostasis with copper ionophores for the treatment of prostate cancer.

### 2.2 Inhibition of Androgen Receptor Leads to Increased Oxidative Phosphorylation

**Figure 2:**
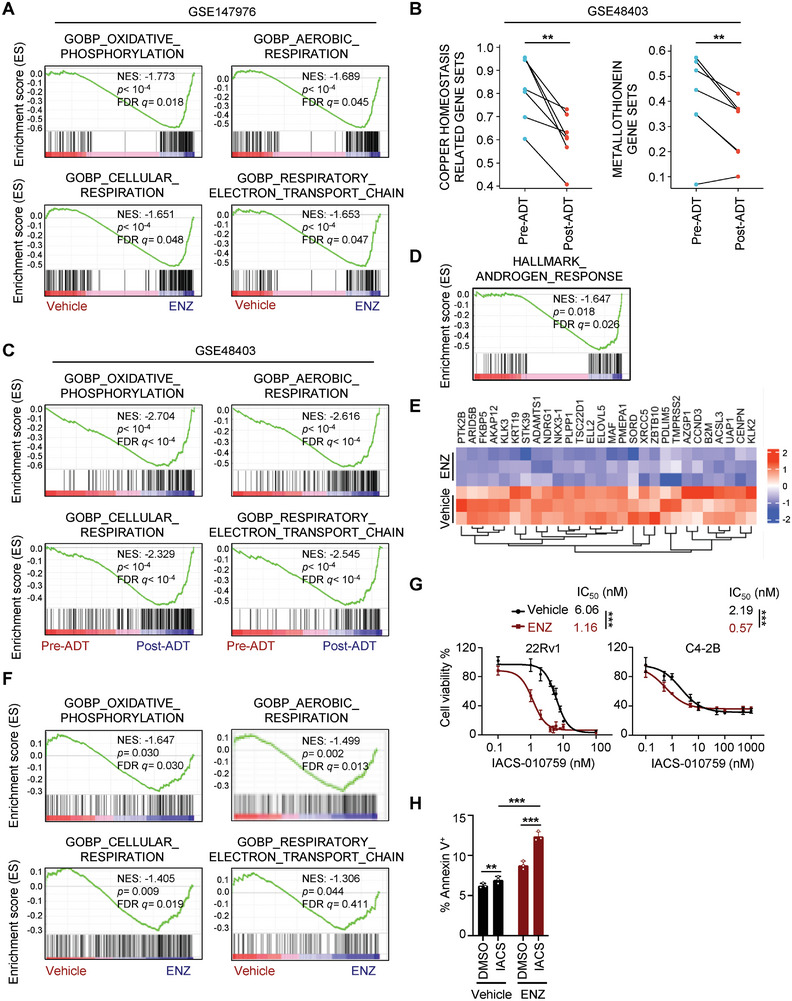
Enzalutamide sensitized prostate cancer cells to inhibition of oxidative phosphorylation. A) Gene Set Enrichment Analysis (GSEA) of gene sets associated with oxidative phosphorylation, aerobic respiration, cellular respiration, and respiration electron transport chain between ENZ‐treated versus vehicle‐treated cells in datasets GSE147976. B) Differences in the expression levels of 12 copper homeostasis‐related genes (left panel) and 12 metallothionein genes (right panel) between matched pre‐ADT and post‐ADT biopsies from the GSE48404 datasets. ^**^ *P* < 0.01 (two‐tailed paired Student's *t*‐test) C) GSEA of gene sets identified in (A) between matched pre‐ADT and post‐ADT tumor samples in datasets GSE48404. D) GSEA of androgen response gene sets between enzalutamide‐treated versus vehicle‐treated 22Rv1 cells after 24‐h treatment with 20 µM ENZ. E) A heatmap depicting the expression levels of AR pathway genes in enzalutamide‐treated and vehicle‐treated 22Rv1 cells after 24‐h treatment with 20 µM ENZ. Row Z‐score values are shown. F) GSEA of the gene sets as shown in (A) in enzalutamide‐treated and vehicle‐treated 22Rv1 cells after 24‐h treatment with 20 µM ENZ. A, C, D, F) Normalized enrichment score (NES), False discovery rate (FDR) q values, and P values of the correlation are shown. G) Cell viability was measured for 22Rv1 and C4‐2B cells after treatment with IACS‐010759 in the presence or absence of enzalutamide for 5 days. 22Rv1 cells: 20 µM ENZ; C4‐2B cells: 10 µM ENZ. Data are shown as Mean ± S.D. for three independent experiments. ^***^ *P* < 0.001 (two‐way ANOVA with Bonferroni's Tukey's multiple comparisons test). H) Apoptotic cell death was determined for 22Rv1 cells treated with 100 nM IACS‐010759 in the presence or absence of 20 µM enzalutamide for 48 h. Data are shown as Mean ± S.D. for three independent experiments. ^**^ *P* < 0.01, ^***^ *P* < 0.001 (two‐tailed unpaired Student's *t*‐test).

While enzalutamide remains a mainstay therapy for CRPC, elucidating its influence on cellular metabolism, particularly mitochondrial dependence, holds potential for uncovering novel therapeutic vulnerabilities.^[^ ^21^ ^]^ We first investigated metabolic changes induced by ENZ‐mediated AR blockade through analysis of the transcriptomic data by Zhang et al.^[^ ^22^ ^]^ dataset. In CWR22 AR‐positive prostate cancer cells, ENZ treatment strongly correlated with gene sets associated with OXPHOS, mitochondrial aerobic respiration, and respiratory electron transport chain (**Figure** **2**A), suggesting potential metabolic vulnerabilities that might influence ENZ sensitivity. To explore these findings in a clinical context, we analyzed the Rajan et al.^[^ ^23^ ^]^ dataset, containing pre‐ and post‐ADT transcriptomic data from seven CRPC patients. Notably, post‐ADT tumors exhibited significant downregulation of copper metabolism and metallothionein genes (Figure 2B), suggesting a potential vulnerability of CRPC to disrupted copper homeostasis following ENZ treatment. Interestingly, our analysis of CRPC tumor data mirrored the CWR22 model, revealing an association between ENZ response and similar gene sets (Figure 2C), further supporting the potential role of metabolic reprogramming in ENZ response. Validating these findings, transcriptomic analysis revealed that ENZ treatment in the 22Rv1 cell line confirmed AR blockade and modulated the expression of gene sets involved in mitochondrial oxidative metabolism, mirroring clinical observations (Figure 2D–F). Importantly, ENZ sensitized CRPC cells to IACS‐010759 (Figure 2G), a mitochondrial complex I inhibitor,^[^ ^24^ ^]^ and potentiated cell death when combined (Figure 2H). Collectively, these results suggest that ENZ treatment increases CRPC dependence on OXPHOS, potentially creating a vulnerability exploitable by copper ionophore‐mediated cell death.

### 2.3 Synergistic Cytotoxic Effects of Copper Ionophores and Enzalutamide in Prostate Cancer Cells

**Figure 3:**
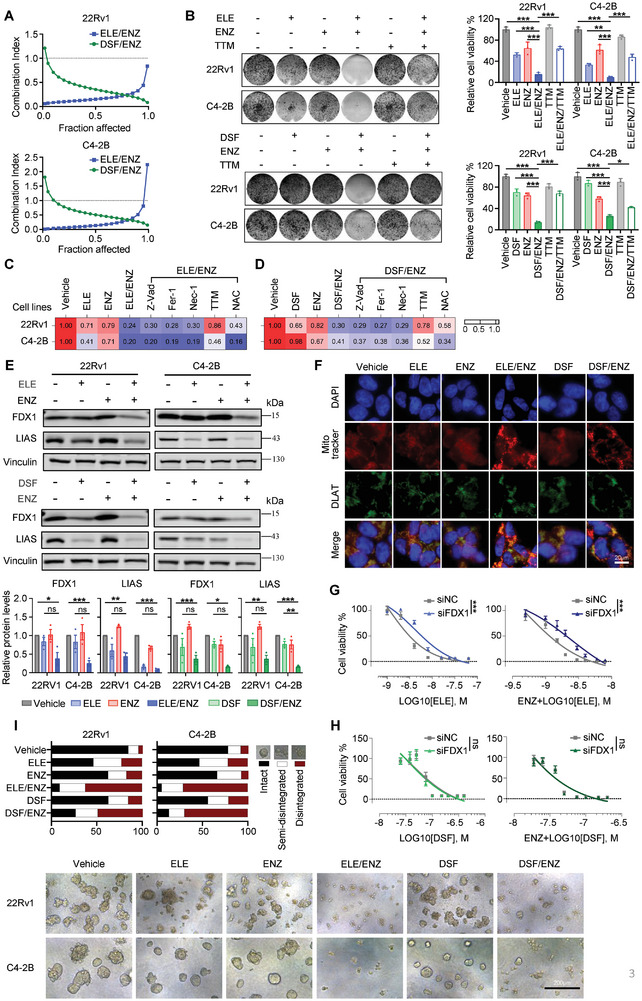
Copper ionophores synergized with enzalutamide to inhibit the growth of prostate cancer cells. A) The synergistic effect of concomitant copper ionophores and enzalutamide in 22Rv1 and C4‐2B cells were measured by crystal violet assay after 5 days of treatment. B) Clonogenic survival of 22Rv1 and C4‐2B cells were analyzed by crystal violet assay after treatment with copper ionophores (ELE or DSF) and/or ENZ for 10 days. 22Rv1 and C4‐2B cells were pretreated with or without 2 µM TTM overnight before exposure to drug treatment. 22Rv1 cells: ELE, 2 nM; DSF, 50 nM; ENZ, 20 µM. C4‐2B cells: ELE, 5 nM; DSF, 100 nM; ENZ, 10 µM. Heatmap depicts viability of 22Rv1 and C4‐2B cells pretreated with 30 µM Z‐VAD‐FMK (Z‐Vad), 10 µM ferrostatin‐1 (Fer‐1), 20 µM necrostatin‐1 (Nec‐1), 20 µM TTM, 2 mM N‐acetylcysteine (NAC) followed by ENZ and/or C) ELE or D) DSF for 5 days. E) Western blot showing expression of FDX1 and LIAS in 22Rv1 and C4‐2B cells following treatment with copper ionophores (ELE or DSF) and/or ENZ for 48 h. Vinculin was used as a loading control. B, E) Data are shown as Mean ± S.D. for three independent experiments. ns, not significant, ^*^ *P* < 0.05, ^**^ *P* < 0.01, ^***^ *P* < 0.001 (one‐way ANOVA, with Tukey's multiple comparisons test). F) Immunofluorescence analysis of DLAT (green), Mitotracker (red), and DAPI (blue) in 22Rv1 cells following treatment with copper ionophores (ELE or DSF) and/or ENZ for 48 h. Scale bars, 20 µm. G, H) Cell viability was measured for 22Rv1 and C4‐2B cells transfected with siNC or siFDX1 following treatment with copper ionophores (ELE or DSF) and/or ENZ. Data are shown as Mean ± S.D. for three independent experiments. ns, not significant, ^***^ *P* < 0.001 (two‐way ANOVA with Bonferroni's Tukey's multiple comparisons test). I) 3D Matrigel cultures of 22Rv1 and C4‐2B cells were treated with copper ionophores (ELE or DSF) and/or ENZ for 10 days. Representative images and quantification of scored structures (intact, semi‐disintegrated, and disintegrated) are shown. 22Rv1 cells: ELE, 5 nM; DSF, 200 nM; ENZ, 20 µM. C4‐2B cells: ELE, 10 nM; DSF, 300 nM; ENZ, 10 µM. Scale bar, 200 µm.

We investigated the synergistic effects of copper ionophores ELE and DSF with ENZ in androgen receptor‐positive CRPC cell lines 22Rv1 and C4‐2B. Both ELE/ENZ and DSF/ENZ combinations synergistically inhibited CRPC cell growth compared to monotherapy groups (Figure S1, Supporting Information), as evidenced by combination indices (CIs) below 1.0 (**Figure** **3**A), and further corroborated by clonogenic survival assays demonstrating superior growth inhibition (Figure 3B). Notably, the copper ion chelator TTM completely reversed the inhibitory effects of these combinations (Figure 3B), suggesting that copper accumulation mediated the synergistic cell death mediated by copper ionophores and ENZ in inducing CRPC cells.

To unravel the underlying mechanism of copper ionophores/ENZ‐mediated cell death in CRPC, we pretreated 22Rv1 and C4‐2B cells with a range of cell death inhibitors, including the apoptosis inhibitor Z‐VAD‐FMK (Z‐Vad), the iron‐mediated cell death inhibitor ferrostatin‐1 (Fer‐1), the necrotic cell death inhibitor necrostatin‐1 (Nec‐1), the ROS inhibitor N‐Acetyl‐L‐cysteine (NAC) and the copper ion chelator TTM. Importantly, only TTM effectively counteracted the synergistic lethality mediated by copper ionophores with ENZ (Figure 3C,D), strongly suggesting a copper‐dependent cell death mechanism. While the ROS inhibitor NAC failed to rescue cells treated with ELE/ENZ, indicating a ROS‐independent mechanism, NAC partially reversed growth inhibition by DSF/ENZ, suggesting a potential involvement of ROS in this combination‐mediated cell death. Further analysis revealed markedly reduced expression of iron‐sulfur cluster proteins FDX1 (mitochondrial ferrodoxin 1) and LIAS (lipoic acid synthetase) in ELE/ENZ‐treated cells compared to controls, ELE‐treated or DSF‐treated alone (Figure 3E), indicating a potential disruption of iron‐sulfur cluster protein stability by the copper ionophores‐ENZ combination. Immunofluorescence staining confirmed an accumulation of DLAT protein aggregates in ELE/ENZ and DSF/ENZ‐treated cells, with a more pronounced effect in the ELE/ENZ group (Figure 3F). Interestingly, ablating the expression of FDX1 through siRNA‐mediated knockdown or CRISPR‐mediated editing, a direct target of the copper ionophore ELE involved in Cu (II) reduction to Cu (I),^[^ ^25^ ^]^ significantly reversed ELE/ENZ‐mediated cell death (Figure 3G; Figure S2, Supporting Information) but minimally affected DSF sensitivity (Figure 3H), indicating distinct cuproptosis mechanisms for these copper ionophores. To further evaluate the in vivo relevance of these findings, we employed 3D matrigel spheroid formation assays. The combination of copper ionophores and ENZ triggered extensive spheroid disintegration in both 22Rv1 and C4‐2B cells, compared to single‐agent treatments (Figure 3I). Collectively, these data provide compelling evidence that copper ionophores and ENZ synergistically induce copper‐dependent cell death in CRPC cells, offering a promising therapeutic strategy for this aggressive cancer.

### 2.4 Synergistic Cytotoxic Effects of Copper Ionophores and Enzalutamide in the Xenograft Mouse Model of Prostate Cancer

**Figure 4:**
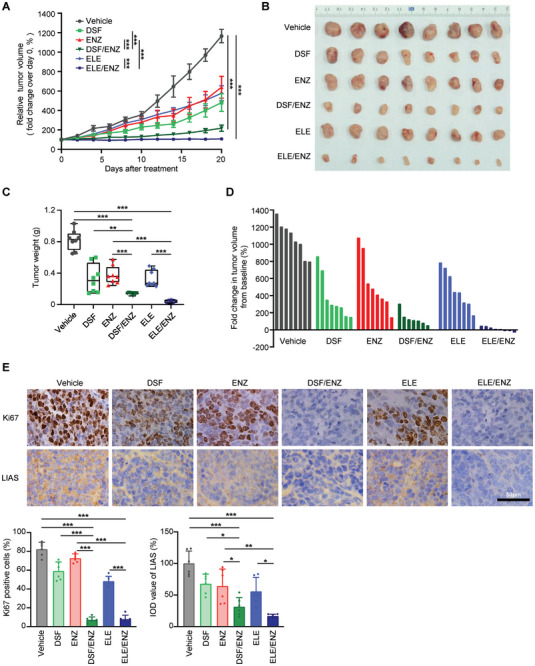
Combined use of copper ionophores and enzalutamide effectively treated xenograft mouse model of CRPC. A) Mice bearing 22Rv1‐xenograft tumors were treated with ENZ and/or copper ionophores ELE or DSF. The graph shows the fold change in tumor volume, with respect to the initial treatment at day 0. n = 8 per treatment group. Data are shown as Mean ± S.E.M. ^***^ *P* < 0.001 (two‐way ANOVA with Bonferroni's Tukey's multiple comparisons test). B) Gross images of the tumors isolated from different treatment groups at the end point. C) Average tumor weights from different treatment groups at the endpoint. D) The waterfall plot indicates fold changes in tumor volume in mice treated as indicated. Fold changes were calculated by (endpoint tumor volume‐baseline) / baseline, multiplied by 100%. Baseline, tumor volume at treatment Day 0. E) Representative images of immunohistochemical staining for Ki67 and LIAS in 22Rv1‐xenograft tumors from different treatment groups at the endpoint. n = 6. IOD, integrated optical density. Scale bars, 50 µm. C,E) Data are shown as the mean ± S.E.M. ^*^ *P* < 0.05, ^**^ *P* < 0.01, ^***^ *P* < 0.001 (one‐way ANOVA, with Tukey's multiple comparisons test).

Our in vitro studies revealed that ENZ reprograms CRPC cells to heavily rely on oxidative phosphorylation for energy, sensitizing them to copper ionophores ELE and DSF. To translate these findings into a preclinical setting, we established a mouse xenograft tumor model using CRPC cells 22Rv1. Tumor‐bearing mice were treated with ELE or DSF monotherapy, ENZ monotherapy or combination therapy with ELE/ENZ or DSF/ENZ. Both ELE and DSF monotherapies significantly suppressed tumor growth compared to controls (**Figure** **4**A–C). However, the combination therapies demonstrated superior antitumor activity, with the ELE/ENZ combination exhibiting the most potent effect, even inducing tumor regression in some cases (Figure 4A–D). Immunohistochemistry revealed a significant decrease in Ki67 staining, a marker of cell proliferation, in both ELE/ENZ and DSF/ENZ combination groups compared to monotherapies (Figure 4E), indicating effective suppression of tumor cell proliferation. Furthermore, we observed a significant downregulation of LIAS, a key enzyme in mitochondrial lipoid acid synthesis,^[^ ^26^ ^]^ in tumor cells from combination therapy groups (Figure 4E), providing further evidence supporting the connection between copper‐mediated cell death and LIAS instability, as previously reported.^[^ ^9^ ^]^ Collectively, these results provide compelling preclinical evidence that the combination of copper ionophores with ENZ represents a promising therapeutic strategy for CRPC.

### 2.5 Synergistic Cytotoxic Effects of Copper Ionophores and Enzalutamide in *Pten p53* Deficient Mouse Prostate Organoids

**Figure 5:**
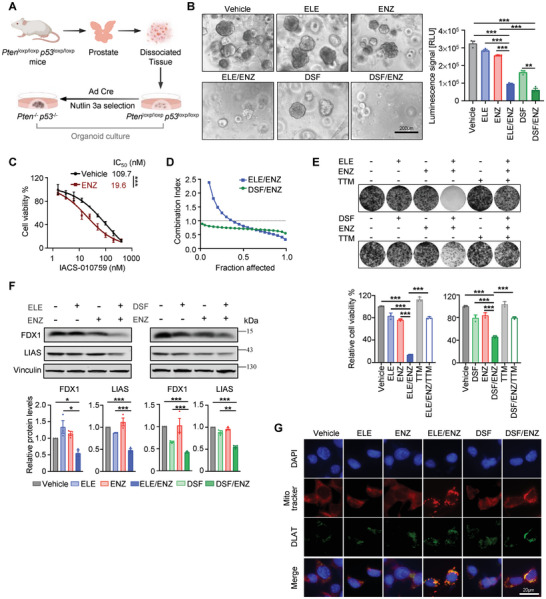
Synergistic cytotoxic effects of copper ionophores and enzalutamide in *Pten p53* deficient mouse prostate organoids. A) Schematics illustrating organoids derived from *Pten* ^loxp/loxp^ *p53* ^loxp/loxp^ mouse prostate tissue. Deletion of *Pten* and *p53* was achieved through adenoviral Cre (Ad‐Cre) infection and Nutlin‐3a selection. B) Representative phase‐contrast images of organoids cultured on Day 4. Scale bar, 200 µm. ATP levels (Luminescence signal) of organoid treated with copper ionophores (ELE, 5 nM) or DSF, 200 nM) and/or ENZ (10 µM) were shown. C) Cell viability was measured by crystal violet assay of MPOd cells treated with IACS‐010759 in the presence or absence of ENZ (5 µM) for 5 days. Cells derived from *Pten* ^−/−^ *p53* ^−/−^ organoids were designated as MPOd. Data are shown as Mean ± S.D. for three independent experiments. ^***^ *P* < 0.001 (two‐way ANOVA with Bonferroni's Tukey's multiple comparisons test). D) The synergistic effect of concomitant copper ionophores and ENZ in MPOd cells was measured by cell viability assay after 5 days treatment. E, Clonogenic survival of MPOd cells treated with copper ionophores (8 nM ELE or 80 nM DSF) and/or 5 µM ENZ was analyzed. MPOd cells were pretreated with or without 2 µM TTM overnight before exposure to drug treatment. F) Western blot showing expression of FDX1 and LIAS in MPOd cells treated with copper ionophores (ELE or DSF) and/or ENZ for 48 h. Vinculin was used as a loading control. B,E,F) Data are shown as mean ± S.D. ^*^ *P* < 0.05, ^**^ *P* < 0.01, ^***^ *P* < 0.001 (one‐way ANOVA, with Tukey's multiple comparisons test). G) Immunofluorescence staining analysis was performed on MPOd cells for DLAT (green), Mitotracker (red), and DAPI (blue) after treatment with copper ionophores (ELE or DSF) and/or ENZ for 48 h. Scale bars, 20 µm. The schematic in A) was created on Biorender.com.

Prostate cancer organoids lacking both *Pten* and *p53* faithfully recapitulating the aggressive features of advanced diseases,^[^ ^27^, ^28^ ^]^ proving a valuable platform for evaluating novel therapeutic strategies. We therefore established a *Pten p53* double knockout (*Pten^−/‐^ p53^−/−^*) organoid line derived from prostate epithelial cells of *Pten* ^loxp/loxp^ *p53* ^loxp/loxp^ mice (**Figure** **5**A **;** Figure S3A,B, Supporting Information). While ENZ alone displayed modest antiproliferative effects on these organoids, its combination with either of the copper ionophores ELE or DSF resulted in a significantly greater inhibitory impact on organoid growth (Figure 5B). This synergistic effect strengthens the notion that ENZ treatment reprograms cancer cells to rely heavily on oxidative phosphorylation for energy. Notably, ENZ treatment further sensitized MPOd (designated from *Pten* ^−/‐^ *p53* ^−/−^ mouse prostate organoid derived) cells to the specific OXPHOS inhibitor IACS‐010759 (Figure 5C), confirming this metabolic shift.

To further explore potential synergistic interactions, we evaluated the combined effects of ENZ and copper ionophores on MPOd cells. Both ELE/ENZ and DSF/ENZ combinations synergistically inhibited the growth of MPOd cells compared to single‐agent treatment, as evidenced by combination indices (CIs) below 1.0 (Figure 5D; Figure S3C, Supporting Information) and further corroborated by clonogenic survival assays demonstrating superior growth inhibition (Figure 5E). The copper chelator TTM effectively reversed the growth inhibition mediated by combination therapy (Figure 5E), confirming the copper‐dependent nature of cell death. Notably, we observed a significant downregulation of FDX1 and LIAS protein levels (Figure 5F), both crucial for iron‐sulfur cluster biogenesis and known to be destabilized during cuproptosis.^[^ ^6^ ^]^ Furthermore, immunofluorescence staining revealed marked aggregation of DLAT, a specific cuproptosis marker, in cells treated with combination therapy (Figure 5G), providing further evidence for distinct cell death pathway.

### 2.6 Synergistic Cytotoxic Effects of Copper Ionophores and Enzalutamide in Enzalutamide‐Resistant Models of CRPC Cancer Cells

**Figure 6:**
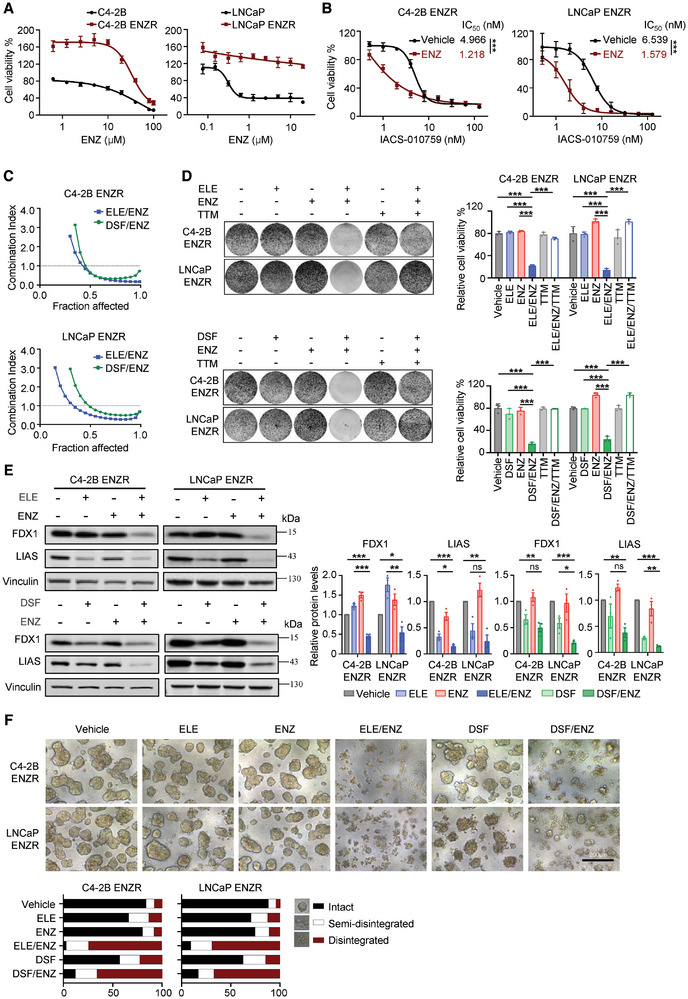
Synergistic cytotoxic effects of copper ionophores and enzalutamide in Enzalutamide‐resistant prostate cancer cells. A) Cell viability was measured by crystal violet assay for C4‐2B and LNCaP parental or ENZR cells treated with ENZ for 5 days. B) Cell viability was measured by crystal violet assay for C4‐2B ENZR and LNCaP ENZR cells treated with IACS‐010759 in the presence or absence of 10 µM ENZ for 5 days. A,B) Data are shown as Mean ± S.D. for three independent experiments. ^***^ *P* < 0.001 (two‐way ANOVA with Bonferroni's Tukey's multiple comparisons test). C) The synergistic effect of concomitant copper ionophores (ELE/DSF) and ENZ in C4‐2B ENZR and LNCaP ENZR cells was measured by crystal violet assay after 5 days of treatment. D) Clonogenic survival of C4‐2B ENZR and LNCaP ENZR cells treated with copper ionophores (ELE, 2 nM, or DSF, 120 nM) and/or ENZ (10 µM) for 7 days was analyzed. C4‐2B ENZR and LNCaP ENZR cells were pretreated with or without 2 µM TTM overnight before exposure to drug treatment. E) Western blot showing expression of FDX1 and LIAS in C4‐2B ENZR and LNCaP ENZR cells after treatment with ELE/DSF and/or ENZ for 48 h. Vinculin was used as a loading control. D,E) Data are shown as Mean ± S.D. for three independent experiments. ns, not significant, ^*^ *P* < 0.05, ^**^ *P* < 0.01, ^***^ *P* < 0.001 (one‐way ANOVA, with Tukey's multiple comparisons test). F) 3D Matrigel cultures of C4‐2B ENZR and LNCaP ENZR cells were treated with ELE (5 nM) / DSF (250 nM) and/or ENZ (20 µM) for 10 days. Representative images and quantification of scored structures (intact, semi‐disintegrated, and disintegrated) are shown. Scale bar, 200 µm.

To evaluate the broader clinical relevance of our findings, we established ENZ‐resistant (ENZR) models of the C4‐2B and LNCaP cell lines (**Figure** **6**A). Notably, these ENZR cells exhibited significantly increased sensitivity to the OXPHOS inhibitor IACS‐010759 following ENZ treatment (Figure 6B), suggesting a heightened dependence on OXPHOS for survival after ENZ exposure. We further hypothesized that ENZ‐induced metabolic reprogramming might sensitized cells to cuproptosis. To test this, we assessed the response of ENZR cells to ENZ and copper ionophores (ELE and DSF) as single‐agents or in combination. Using median‐effect analysis via CalcuSyn, we observed synergistic cytotoxic effects in both C4‐2B and LNCaP ENZR cells (Figure 6C; Figure S4, Supporting Information). Clonogenic survival assays confirmed synergistic growth inhibition by the combination (Figure 6D). Importantly, the copper chelator TTM effectively reversed this synergistic effect (Figure 6D), providing compelling evidence for its copper‐dependent nature. Moreover, the combination therapy significantly downregulated iron‐sulfur cluster proteins FDX1 and LIAS in ENZR cells (Figure 6E), consistent with the established hallmarks of copper‐mediated death,^[^ ^9^, ^10^, ^29^ ^]^ characterized by accumulation of copper‐bound proteins and loss of iron‐sulfur cluster proteins. Notably, the copper ionophores/ENZ combination induced extensive spheroid disintegration in both C4‐2B and LNCaP ENZR cells compared to single‐agent treatments (Figure 6F). Collectively, these data suggest that ENZ‐resistant CRPC cells exhibit an increased reliance on OXPHOS, creating a vulnerability exploitable by combined therapeutic strategies targeting ENZ‐ and copper ionophores‐mediated cell death pathways.

## 3 Discussion

Cuproptosis, a recently discovered cell death mechanism intimately associated with oxidative phosphorylation and the lipoic acid pathway,^[^ ^9^, ^10^ ^]^ shows significant potential as an anti‐cancer therapeutic approach. Our findings indicate the specific inhibitory effects of copper ionophores on prostate cancer cells compared to normal cells, suggesting their potential as therapeutic agents. Furthermore, we have demonstrated a synergistic suppression of cancer cell growth through the combined use of enzalutamide and copper ionophores. This synergistic effect, characterized by aggregation of lipoylated proteins and instability of iron‐sulfur cluster proteins, manifests as cuproptosis, a form of copper‐dependent cell death distinct from other types of cell death related to oxidative stress (e.g., apoptosis, ferroptosis and necroptosis).

Metabolic reprogramming, particularly the shift towards increased dependence on specific metabolic pathways, has become a recognized Achilles' heel of therapy‐resistant cells.^[^ ^30^, ^31^ ^]^ This phenomenon offers exciting new avenues for overcoming treatment resistance, potentially leading to significantly improved responses in patients facing challenges with conventional therapies. Our study, focused on castration‐resistant prostate cancer, specifically identified metabolic reprogramming toward enhanced reliance on OXPHOS as a key vulnerability for overcoming resistance to the common drug enzalutamide, paving the way for novel therapeutic strategies that target these metabolic dependencies. Our findings align with previous studies that indicate AR inhibition reprograms cellular metabolism to rely more on oxidative phosphorylation,^[^ ^16^, ^17^ ^]^ making CRPC cells more susceptible to copper ionophores. This convergence suggests a broader principle that AR‐targeting drugs may universally promote dependence on OXPHOS, thereby increasing sensitivity to copper‐mediated cell death. As a result, combination therapy regimens incorporating copper ionophores alongside AR‐targeting drugs may offer increased flexibility and potentially broader efficacy in combating prostate cancer. Interestingly, ENZ‐resistant cells exhibited accelerated proliferation in the presence of ENZ and showed sensitivity to the oxidative phosphorylation inhibitor IACS‐010759 only when co‐administered with ENZ. This suggests that androgen receptor inhibition paradoxically enhances oxidative phosphorylation and promotes cellular proliferation. As a result, timely discontinuation of drug treatment upon emergence of resistance during AR inhibitor therapy could potentially inhibit tumor growth. Alternatively, the combination of copper ionophores with ongoing drug administration may represent an additional approach to synergistically enhance tumor cell eradication.

Understanding the diverse mechanisms of copper‐mediated cell death is essential for harnessing its potential as a targeted cancer therapy. Our findings indicate that FDX1 knockdown significantly impairs sensitivity to ELE, a known FDX1‐dependent copper ionophore, while having minimal impact on DSF‐mediated cell death. This suggests distinct mitochondrial copper release pathways for these agents.^[^ ^9^, ^13^ ^]^ This observation is supported by emerging evidence that bis‐diethyldithiocarbamate‐copper (CuET), the copper‐containing metabolite of DSF, induces NPL4 (a subunit of the p97/VCP segregase) aggregation‐mediated cytotoxicity independently of its established ALDH inhibitory activity.^[^ ^32^, ^33^ ^]^ Indeed, our further experiments demonstrated that the combined use of CuET and ENZ also significantly inhibited the growth of 22Rv1 and MPOd cells (Figure S5, Supporting Information), mirroring the combinatorial cytotoxic effects of DSF and ENZ. Given that CuET does not inhibit ALDH activity,^[^ ^32^ ^]^ the synergistic effect of ENZ and DSF in CRPC cells appears to be independent of ALDH inhibition. Elucidating the precise mechanisms governing FDX1‐dependent and FDX1‐independent copper release from different copper ionophores, including the interaction of DSF with NPL4, is critical for a comprehensive understanding of copper‐mediated cell death. Utilizing FDX1 knockout mouse models, conducting comparative analyses of intracellular copper localization, and exploring mitochondrial copper transport pathways in detail may provide valuable insights to resolve these discrepancies and advance our knowledge of copper‐mediated cell death.

Taken together, these findings highlight the synergistic induction of cuproptosis by copper ionophores and enzalutamide, unveiling a promising therapeutic avenue for CRPC, potentially including cases exhibiting resistance to enzalutamide treatment.

## 4 Experimental Section

### Cell Culture and Reagents

DU145, PC‐3, 22Rv1, LNCaP, C4‐2B, and RWPE‐1 were obtained from the American Type Culture Collection (ATCC). DU145 cells were maintained in the DMEM medium (Gibco). PC‐3, 22Rv1, LNCaP, C4‐2B and RWPE‐1 cells were maintained in RPMI1640 medium (Gibco). All cells were cultured supplemented with 10% fetal bovine serum (FBS, Biological Industries) and 100 units/ml penicillin/streptomycin (Gibco) at 37 °C in a humidified incubator with 5% CO_2_. All cell lines were authenticated using short tandem repeat (STR) profiling and tested periodically for mycoplasma contamination at the beginning of this study.

Enzalutamide (HY‐70002), Elesclomol (HY‐12040), Disulfiram (HY‐B0240), IACS‐010759 (HY‐112037), Z‐VAD‐FMK (HY‐16658B), Necrostatin‐1 (HY‐15760), Ferrostatin‐1 (HY‐100579) and Nutlin‐3a (HY‐10029) were purchased from MedChemExpress (MCE). Copper (II) chloride dihydrate (307 483), Tetrathiomolybdate (323 446), and N‐Acetyl‐L‐cysteine (A9165) were purchased from Sigma–Aldrich. Bis‐diethyldithiocarbamate‐copper (C154088) was purchased from Aladdin.

siRNAs were custom synthesized from GenePharma. Cells were transfected with on‐target (siFDX1, 5′‐ATGGACAATATGACTGTTCGA‐3′) or non‐targeting control siRNA (siNC, 5′‐UUCUCCGAACGUGUCACGUTT‐3′) using Lipofectamine 3000 (Invitrogen, L3000150) according to the manufacturer's protocols.

### Plasmids

To construct LentiCrisprV2 (Addgene ^#^52 961) targeting FDX1, synthesis of the sequence‐specific oligonucleotides was performed by Sangon Biotech. The sequence was referred to Tsvetkov, et al.^[^ ^9^ ^]^

### Establishment of Enzalutamide‐Resistant (ENZR) Cells

Enzalutamide‐resistant cells were generated according to the protocol described previously with minor modifications.^[^ ^34^, ^35^ ^]^ The AR‐positive prostate cancer cell lines LNCaP and C4‐2B were subjected to chronic exposure to increasing concentrations of enzalutamide over an eight‐month period. Briefly, the parental LNCaP and C4‐2B cells were initially cultured in RPMI1640 culture medium containing enzalutamide at concentrations slightly below their respective IC50 (LNCaP, 0.1 µm; C4‐2B, 5 µm) until they reached ≈ 90% confluence. The cells were then sub‐cultured and continuously exposed to escalating doses of enzalutamide, reaching a maximum of 40 µm. Finally, the cell lines that demonstrated exponential growth in the presence of 10 µm enzalutamide were designated as Enzalutamide‐resistant (ENZR) cells.

### Mouse Prostate Organoid Culture

*Pten* ^loxp/loxp^ *p53* ^loxp/loxp^ mice, fully backcrossed to the FVB background, were obtained under a materials transfer agreement from Dana Farber Cancer Institute, Harvard Medical School. All animal procedures were approved by the approval of the Animal Care and Use Committee at Dalian Medical University. Eight‐week‐old male *Pten* ^loxp/loxp^ *p53* ^loxp/loxp^ mice underwent prostate dissection using a dissecting microscope. Subsequently, 3D organoids were generated from the prostate tissue as described previously.^[^ ^36^, ^37^ ^]^ Briefly, surgically excised prostate tissues were dissected into small pieces and digested with Collagenase Type II (Gibco) at 37 °C. Following centrifugation, tissue pellets were resuspended in AdDF+++ [Advanced DMEM/F12 supplemented with 1% HEPES (Life Technologies, 15 630 080), 1 x GlutaMAX (Life Technologies, 35 050 061) and antibiotics] and pipetted repeatedly with a syringe before filtration through a 100 µm filter. Dissociated cells were then washed and seeded in Growth Factor‐reduced Matrigel (BD Biosciences, 356 231), cultured in AdDF+++ containing various growth factors and inhibitors as described,^[^ ^36^, ^37^ ^]^ including Noggin (PeproTech, 250‐38), Rspo1 (PeproTech, 315–32), N‐Acetylcysteine (Sigma–Aldrich, A9165), A83‐01 (Sigma‐Aldrich, SML0788), FGF10 (PeproTech, 100–26), Y‐27632 (AbMole Bioscience, M20999), EGF (PeproTech, 100–15) and B27 supplement (Life Technologies, 17 504 044). Mouse prostate organoid *Pten* and *p53* deletion was achieved through adenovirus Cre (Ad‐Cre) infection and 10 µm Nutlin 3a selection.

### Cell Viability Assay

Cells seeded in 96‐well plates were exposed to drug treatment conditions as specified. Freshly prepared drug‐containing medium was provided every other day for continued exposure. Cells were fixed and stained with 0.5% crystal violet solution. Bound crystal violet was resolved by 50% acetic acid solution. The optical absorbance (OD) (at 570 nm) of bound crystal violet was measured using xMark Microplate Absorbance Spectrophotometer (Bio‐Rad Laboratories). The IC50 values were calculated using GraphPad Prism software. The synergy effect was calculated by the Chou‐Talalay method to calculate the combination index (CI).

In the organoid viability assay, organoids were cultured in 96‐well plates followed by drug exposure as indicated. Fresh medium was replaced every other day. The ATP levels were measured with CellTiter‐Glo 3D Reagent (Promega, G9681) in accordance with the manufacturer's instructions, followed by luminescence signal detection on a SpectraMax microplate reader (Molecular Devices).

### Clonogenic Survival Assay

Cells were seeded in 24‐well culture plates and replaced with fresh medium with or without drugs every other day. At the endpoint, cells were fixed with 0.5% crystal violet solution and dissolved with 50% glacial acetic acid solution. The optical density (OD) was measured at 570 nm by an xMARK Microplate Spectrometer (BioRad).

### Flow Cytometry Analysis

Apoptosis was assessed using the Annexin V‐FITC Apoptosis Detection kit (Dojindo Molecular Technologies AD10) according to the manufacturer's protocol. Following treatment with 0.25% trypsin without EDTA, cultured cells were stained with annexin V‐FITC and Propidium iodide (PI) solution.

### 3D Spheroid Assay

3D spheroid culture experiments were conducted as previously described.^[^ ^38^ ^]^ Briefly, cells were seeded on plates pre‐coated with Matrigel (BD Biosciences) and grown in RPMI1640 medium supplemented with 2% FBS and 2% Matrigel and allowed to grow for 3 days before drug exposure. Fresh medium containing 2% FBS and Matrigel was replaced every two days. The 3D structures were imaged by inverted phase contrast microscope (Leica Microsystems, Germany) and scored according to 3D structure integrity. Over 200 structures were scored for each condition.

### Western Blot

Cell lysates were prepared using ice‐cold RIPA buffer supplemented with protease/phosphatase inhibitors (Roche). Western blot experiments were conducted as described previously.^[^ ^39^ ^]^ The following antibodies were used: FDX1 (Abcam, ab108257), LIAS (Proteintech, 11577‐1‐AP) and Vinculin (Sigma, V9131). Western blots were imaged using Odyssey Infrared Imaging System (Li‐COR Biosciences).

### RNA Sequencing Analysis

RNA was isolated from 22Rv1 cells following 24‐h treatment with the indicated using TRIzol Reagent (Invitrogen, 15 596 018). RNA‐Seq was performed by Novogene Corporation. The sequencing libraries were generated using NEBNext UltraTM RNA Library Prep Kit for Illumina according to the manufacturer's instructions. Gene set enrichment analysis (GSEA) was performed using the JAVA program (http://software.broadinstitute.org/gsea/index.jsp) to identify the molecular pathways correlated to Enzalutamide treatment response in 22Rv1 cells. The Molecular Signatures Database (MSigDB) served as the reference database. A heat map was generated using individual genes from the Androgen response pathway, employing the “pheatmap” package. 1000 permutations were carried out to determine statistical significance. Gene sets with a false discovery rate (FDR) ≤0.05 and nominal p‐values ≤0.01 were considered significantly enriched.

### Data Analysis

Processed data from the Toil RNAseq pipeline^[^ ^40^ ^]^ were retrieved from UCSC Xena (https://xenabrowser.net/datapages/), encompassing transcriptomes for 152 normal tissues from GTEx and 496 tumor tissues from TCGA. These data were normalized using Transcripts Per Million (TPM) units. The datasets GSE147976^[^ ^22^ ^]^ and GSE48403^[^ ^23^ ^]^ were downloaded from Gene Expression Ominibus (GEO). Gene Set Enrichment Analysis (GSEA) was performed across the Molecular Signatures Database (MSigDB) using the R package cluster Profiler to identify the molecular pathways correlated with the responses to ADT treatment in prostate cancer.

### In Vivo Mouse Xenograft Study

Eight‐week‐old male NYG mice (Liaoning Changsheng Biotechnology Co., Ltd.) were maintained in a pathogen‐free environment. All animal experiments were performed under the approval of the Animal Research Committee of Dalian Medical University. ≈ 5 × 10^6^ 22Rv1 cells mixed with Matrigel (BD Biosciences) were inoculated into NYG mice subcutaneously. Drug treatment started when tumors reached an average volume of 100 mm^3^. Disulfiram (25 mg kg^−1^) dissolved in 2% DMSO, 40% PEG‐300, 5% Tween‐80, and 53% saline was administered via oral gavage daily. Elesclomol (25 mg kg^−1^) dissolved in 1% DMSO, 40% PEG‐300, 5% Tween‐80, and 54% saline was administered via subcutaneous injection twice a week. Enzalutamide (25 mg kg^−1^) dissolved in a solution of 50% PEG‐300 and 50% Saline was administered via oral gavage every other day. Tumor volumes were measured with calipers and calculated according to the following formula: tumor volume = (length × width^2^) × 2^−1^.

### Quantitative Reverse Transcription PCR (qRT‐PCR)

Total RNA was harvested from cells using SevenFastTotal RNA Extraction Kit for Cells (Seven, SM130‐02) according to the manufacturer's instructions. Reverse transcription reaction was performed using total RNA by the cDNA synthesis kit (Accurate Biotechnology, AG11706) and quantitative PCR was performed using SYBR Select Master Mix (Monad, MQ00401S) on the QuantStudio 5 Real‐Time PCR system (Thermo Fisher). Gene expression was normalized to ACTB. The primers used are shown as follows:

FDX1
5′‐CTTTGGTGCATGTGAGGGAA‐3′ (sense)
5′‐GCATCAGCCACTGTTTCAGG‐3′ (antisense)
ACTB
5′‐CATGTACGTTGCTATCCAGGC‐3′ (sense)
5′‐CTCCTTAATGTCACGCACGAT‐3′ (antisense)

### Immunofluorescence Staining Analysis

Immunofluorescence staining was performed as previously described.^[^ ^41^ ^]^ Cells were incubated with 100 nm Mitotracker (Thermo Fisher, M7512) for 30 min prior to fixation. Fixed cells were stained with DLAT antibody (CST, 12362S) at 4 °C overnight followed by staining with DAPI solution (Sigma–Aldrich, D9542). Fluorescence‐conjugated secondary antibodies were used. Images were photographed with a fluorescence microscope (Leica DM6B Thunder).

### Histology and Immunohistochemical Staining

Tumors were isolated and fixed in formalin over 24 h before paraffin embedding. Paraffin blocks were sectioned and stained with the following primary antibodies: LIAS (Proteintech, 11577‐1‐AP) and Ki67 (Abcam, ab15580). For each tumor sample, 6 random 40 × fields were scored. Protein levels were quantified using ImageJ software.

### Statistics and Reproducibility

Statistical analyses were performed with Wilcoxon rank sum test, two‐tailed paired or unpaired Student's *t*‐test, one‐way ANOVA with Tukey's multiple comparisons test or two‐way ANOVA with Bonferroni's Tukey's multiple comparisons test (GraphPad Prism and SPSS 26). All data shown represent the results obtained from three (or as indicated) independent experiments with the Mean ± S.D. or Mean ± S.E.M. P values < 0.05 were considered statistically significant.

## Conflict of Interest

The authors declare no potential conflicts of interest.

## Author Contributions

X.G., H.Z., and J.L. contributed equally to this work. X.G. performed conceptualization, data curation, formal analysis, methodology, validation, visualization, and wrote–original draft. H.Z. performed data curation, formal analysis, investigation, methodology, and visualization. J.L. performed the investigation and methodology. M.W. performed the investigation and methodology. Z.D. performed data curation and resources. W.H. performed formal analysis and visualization. Y.W. performed data curation and investigation. X.W. performed an investigation. M.Z. performed methodology. P.L. performed conceptualization, funding acquisition, methodology, project administration, resources, and supervision. H.C. performed conceptualization, funding acquisition, methodology, project administration, resources, supervision, wrote–reviewed, and edited. Z.L. performed conceptualization, funding acquisition, supervision, wrote–reviewed, and edited.

## Supporting information

Supporting Information

## Data Availability

The data that support the findings of this study are available from the corresponding author upon reasonable request.
