# Supplementary material for: Enzalutamide Sensitizes Castration‐Resistant Prostate Cancer to Copper‐Mediated Cell Death
Source: Adv Sci (Weinh). 2024 Jun 10;11(30):2401396. doi: 10.1002/advs.202401396 (PMC11321675; doi:10.1002/advs.202401396)
Supplement: Supplementary file 1 — Supporting Information [file ADVS-11-2401396-s001.docx]

Supporting Information

Enzalutamide Sensitizes Castration-Resistant Prostate Cancer to Copper-Mediated Cell Death

Xiang Gao, Haolin Zhao, Jiao Liu, Min Wang, Zhihong Dai, Wenjun Hao, Yanlong Wang, Xiang Wang, Min Zhang, Pixu Liu*, Hailing Cheng*, and Zhiyu Liu*


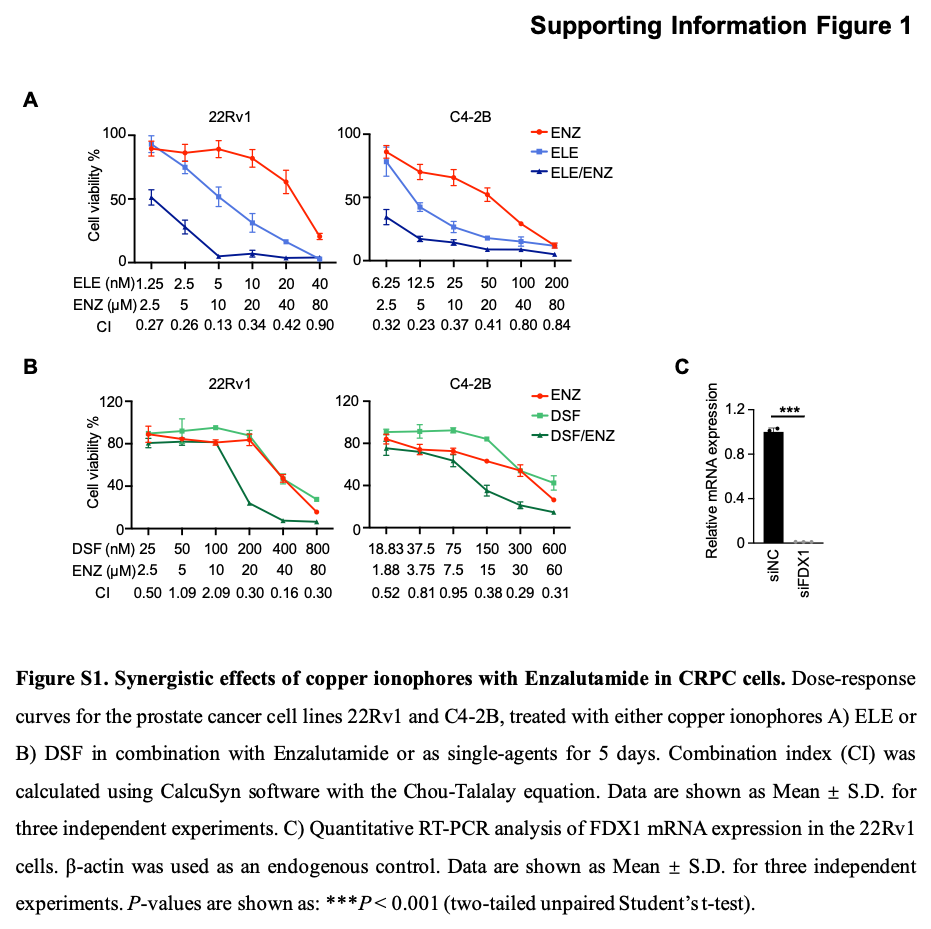


**Figure S1. Synergistic effects of copper ionophores with enzalutamide in CRPC cells.** Dose-response curves for the prostate cancer cell lines 22Rv1 and C4-2B, treated with either copper ionophores A) ELE or B) DSF in combination with enzalutamide or as single-agents for 5 days. Combination index (CI) was calculated using CalcuSyn software with the Chou-Talalay equation. Data are shown as Mean ± S.D. for three independent experiments. C) Quantitative RT-PCR analysis of FDX1 mRNA expression in the 22Rv1 cells. β-actin was used as an endogenous control. Data are shown as Mean ± S.D. for three independent experiments. *P*-values are shown as: ****P* < 0.001 (two-tailed unpaired Student’s t-test).


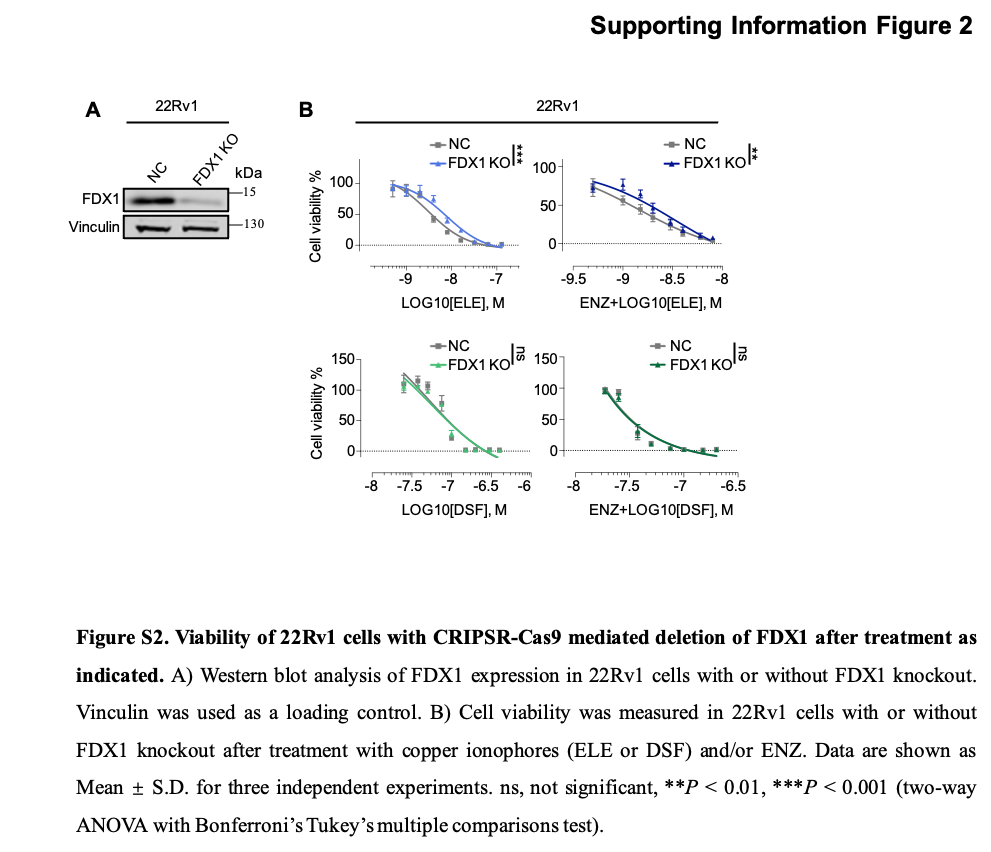


**Figure S2. Viability of 22Rv1 cells with CRIPSR-Cas9 mediated deletion of FDX1 after treatment as indicated.** A) Western blot analysis of FDX1 expression in 22Rv1 cells with or without FDX1 knockout. Vinculin was used as a loading control. B) Cell viability was measured in 22Rv1 cells with or without FDX1 knockout after treatment with copper ionophores (ELE or DSF) and/or ENZ. Data are shown as Mean ± S.D. for three independent experiments. ns, not significant, ***P* < 0.01, ****P* < 0.001 (two-way ANOVA with Bonferroni’s Tukey’s multiple comparisons test).


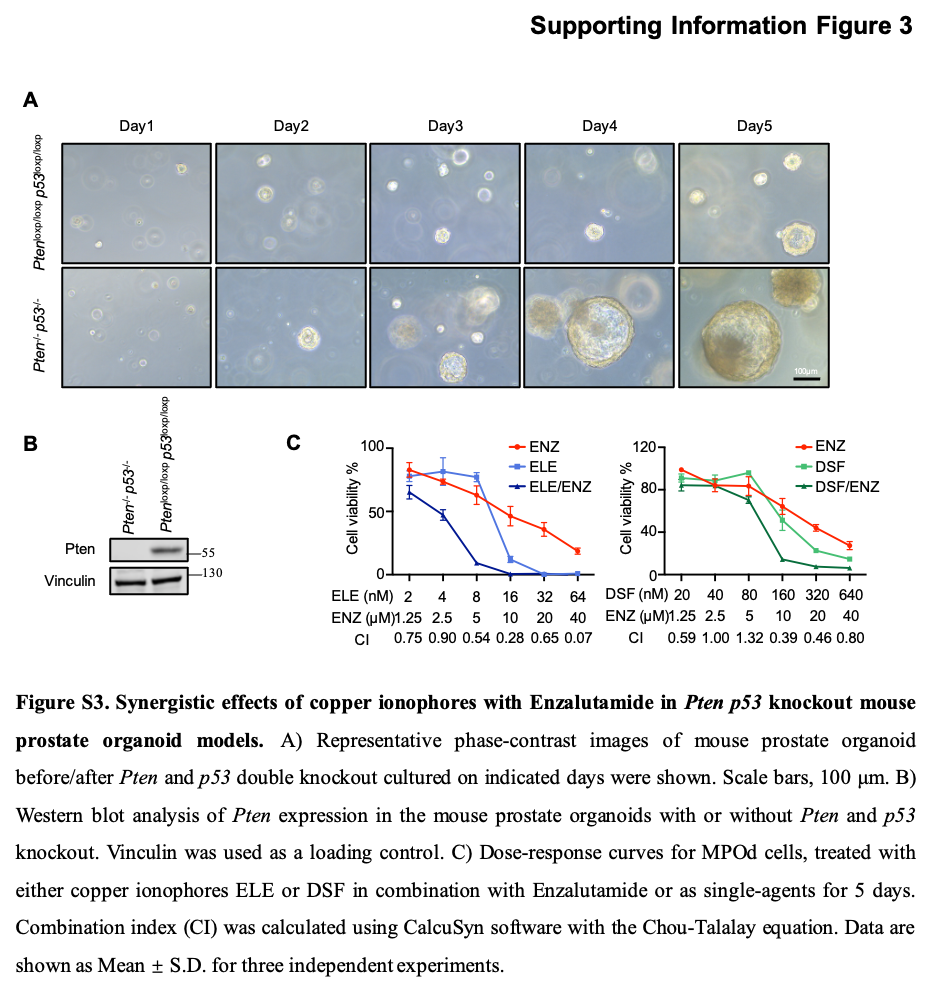


**Figure S3. Synergistic effects of copper ionophores with enzalutamide in *Pten p53* knockout mouse prostate organoid models.** A) Representative phase-contrast images of mouse prostate organoid before/after *Pten* and *p53* double knockout cultured on indicated days were shown. Scale bars, 100 μm. B) Western blot analysis of *Pten* expression in the mouse prostate organoids with or without *Pten* and *p53* knockout. Vinculin was used as a loading control. C) Dose-response curves for MPOd cells, treated with either copper ionophores ELE or DSF in combination with Enzalutamide or as single-agents for 5 days. Combination index (CI) was calculated using CalcuSyn software with the Chou-Talalay equation. Data are shown as Mean ± S.D. for three independent experiments.


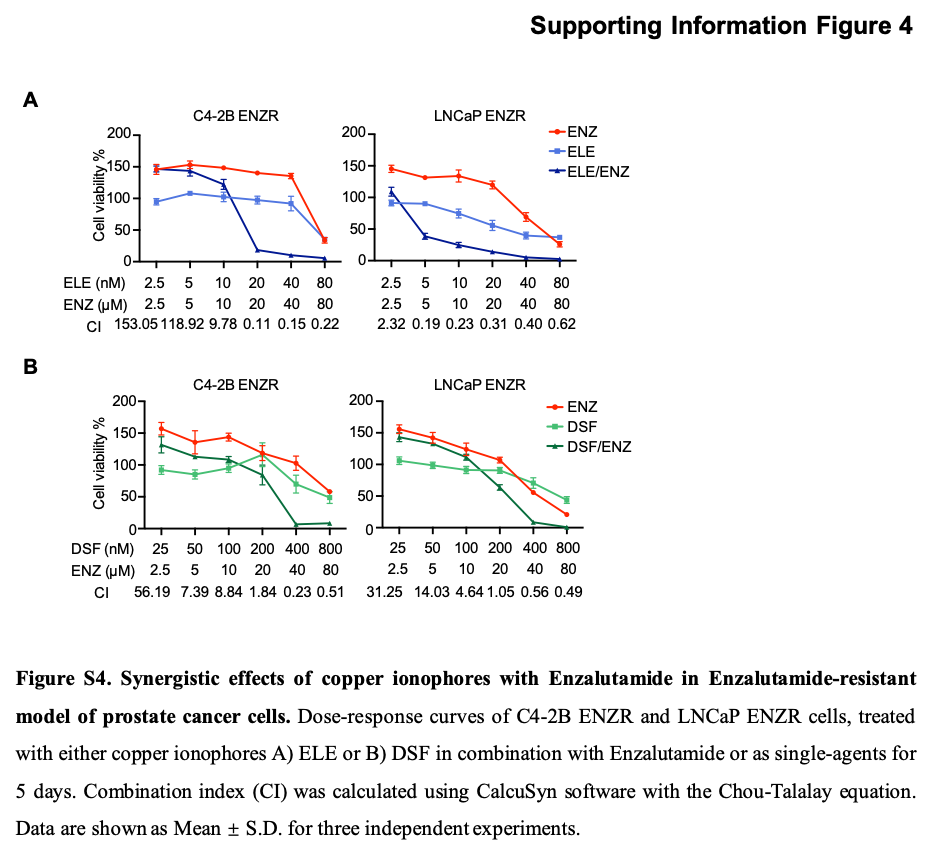


**Figure S4. Synergistic effects of copper ionophores with enzalutamide in Enzalutamide-resistant model of prostate cancer cells.** Dose-response curves of C4-2B ENZR and LNCaP ENZR cells, treated with either copper ionophores A) ELE or B) DSF in combination with enzalutamide or as single-agents for 5 days. Combination index (CI) was calculated using CalcuSyn software with the Chou-Talalay equation. Data are shown as Mean ± S.D. for three independent experiments.


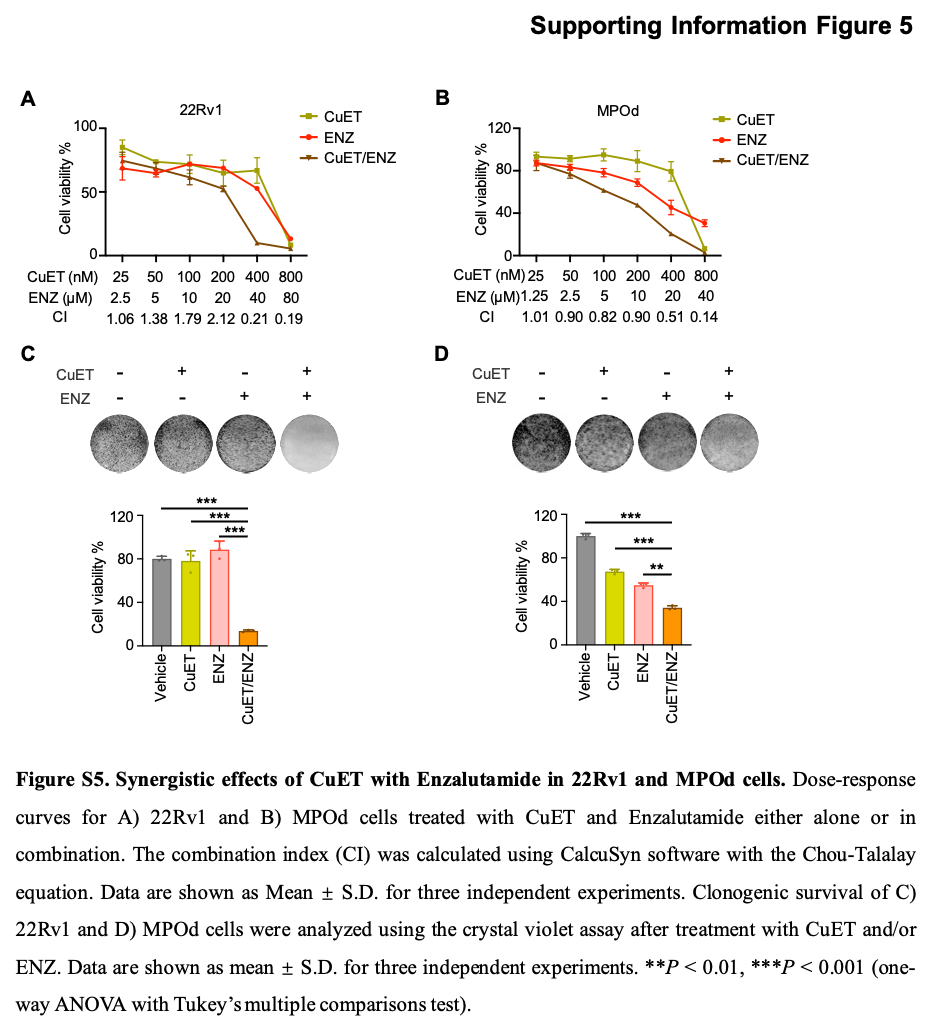


**Figure S5. Synergistic effects of CuET with enzalutamide in 22Rv1 and MPOd cells.** Dose-response curves for A) 22Rv1 and B) MPOd cells treated with CuET and enzalutamide either alone or in combination. The combination index (CI) was calculated using CalcuSyn software with the Chou-Talalay equation. Data are shown as Mean ± S.D. for three independent experiments. Clonogenic survival of C) 22Rv1 and D) MPOd cells were analyzed using the crystal violet assay after treatment with CuET and/or ENZ. Data are shown as mean ± S.D. for three independent experiments. ***P* < 0.01, ****P* < 0.001 (one-way ANOVA with Tukey’s multiple comparisons test).
